# Peptides targeting dengue viral nonstructural protein 1 inhibit dengue virus production

**DOI:** 10.1038/s41598-020-69515-9

**Published:** 2020-07-31

**Authors:** Pucharee Songprakhon, Thanawat Thaingtamtanha, Thawornchai Limjindaporn, Chunya Puttikhunt, Chatchawan Srisawat, Prasit Luangaram, Thanyaporn Dechtawewat, Chairat Uthaipibull, Sissades Thongsima, Pa-thai Yenchitsomanus, Prida Malasit, Sansanee Noisakran

**Affiliations:** 1 Division of Molecular Medicine, Research Department, Faculty of Medicine Siriraj Hospital, Mahidol University, Bangkok, Thailand; 2 Faculty of Pharmacy, Rangsit University, Pathum Thani, Thailand; 3 Department of Anatomy, Faculty of Medicine Siriraj Hospital, Mahidol University, Bangkok, Thailand; 4 Molecular Biology of Dengue and Flaviviruses Research Team, Medical Molecular Biotechnology Research Group, National Center for Genetic Engineering and Biotechnology, National Science and Technology Development Agency, Bangkok, Thailand; 5 Division of Dengue Hemorrhagic Fever Research, Research Department, Faculty of Medicine Siriraj Hospital, Mahidol University, Bangkok, Thailand; 6 Siriraj Center of Research Excellence in Dengue and Emerging Pathogens, Faculty of Medicine Siriraj Hospital, Mahidol University, Bangkok, Thailand; 7 Department of Biochemistry, Faculty of Medicine Siriraj Hospital, Mahidol University, Bangkok, Thailand; 8 Protein-Ligand Engineering and Molecular Biology Research Team, Medical Molecular Biotechnology Research Group, National Center for Genetic Engineering and Biotechnology, National Science and Technology Development Agency, Pathum Thani, Thailand; 9 National Biobank of Thailand, National Science and Technology Development Agency, Pathum Thani, Thailand; 10 Present Address: Department of Chemistry and Biology, University of Siegen, Siegen, Germany

**Keywords:** Microbiology, Virology, Dengue virus

## Abstract

Viruses manipulate the life cycle in host cells via the use of viral properties and host machineries. Development of antiviral peptides against dengue virus (DENV) infection has previously been concentrated on blocking the actions of viral structural proteins and enzymes in virus entry and viral RNA processing in host cells. In this study, we proposed DENV NS1, which is a multifunctional non-structural protein indispensable for virus production, as a new target for inhibition of DENV infection by specific peptides. We performed biopanning assays using a phage-displayed peptide library and identified 11 different sequences of 12-mer peptides binding to DENV NS1. In silico analyses of peptide-protein interactions revealed 4 peptides most likely to bind to DENV NS1 at specific positions and their association was analysed by surface plasmon resonance. Treatment of Huh7 cells with these 4 peptides conjugated with N-terminal fluorescent tag and C-terminal cell penetrating tag at varying time-of-addition post-DENV infection could inhibit the production of DENV-2 in a time- and dose-dependent manner. The inhibitory effects of the peptides were also observed in other virus serotypes (DENV-1 and DENV-4), but not in DENV-3. These findings indicate the potential application of peptides targeting DENV NS1 as antiviral agents against DENV infection.

## Introduction

Dengue virus (DENV) infection is a major and increasing public health problem worldwide. There are approximately 390 million infections, 500,000 severe cases with hospitalization, and a 2.5% mortality rate each year^1,2^. Although the majority of individuals experiencing DENV infection are asymptomatic, approximately one-fourth of the infected cases develop a wide range of clinical manifestations of dengue disease with unclear pathogenic mechanisms^3,4^. To date, there is no specific anti-viral drug available for the treatment of DENV infection. A licensed dengue vaccine also has some limitations for use in naïve individuals not previously infected by DENV and in children less than 9 years of age ^5,6^. Consequently, the development of an alternative strategy to combat DENV infection and severe dengue is still needed.

DENV is a positive, single-stranded, enveloped RNA virus belonging to the family *Flaviviridae*, and it has 4 distinct serotypes (DENV-1, DENV-2, DENV-3 and DENV-4)^7^. Infection with any DENV serotype can generate 10 viral protein products that serve as structural components (capsid, C; pre-membrane, prM; envelope, E) and nonstructural components (NS1, NS2A, NS2B, NS3, NS4A, NS4B, and NS5) of DENV, and each exerts different functions in host cells^7,8^. NS1 is a key viral protein that is required for both DENV replication and dengue disease pathogenesis. The NS1 protein comprises of 352 amino acid residues that share approximately 70% sequence similarity among all four DENV serotypes and 40–50% sequence similarity to other flaviviruses^9,10^. The NS1 protein is initially synthesized in DENV-infected cells as a monomeric form, which subsequently undergoes glycan modifications and dimerization^11,12^. Dimeric NS1 associates with host cell membrane via lipid rafts and glycosylphosphatidylinositol linkage and is detectable on the surface of virus-infected cells^13,14^. In addition, the NS1 protein can be oligomerized and secreted into the extracellular milieu in the form of soluble hexameric lipoprotein^15,16^.

The DENV NS1 protein has three important domains, including hydrophobic β-roll (dimerization domain, amino acids 1–29), wing (connector subdomains, amino acids 30–37 and 152–180; α/β subdomain, amino acids 38–151), and β-ladder (amino acids 181–352), which play roles in viral RNA replication, DENV production, and modulation of host immune responses^17–19^. In DENV-infected cells, NS1 protein is required for the formation of virus-induced membrane structures as the sites of DENV replication ^20^. It colocalizes with double-stranded viral RNA ^21^ and interacts with NS4A-2K-NS4B precursor and DENV structural proteins (C, prM and E) that are essential for viral RNA replication and production of infectious virions, respectively^19,20^. Intracellular NS1 also interacts with certain human cellular proteins that are involved in DENV replication and dengue pathogenesis^22–27^. NS1 protein on the cell surface can trigger complement activation and intracellular signaling upon crosslinking with specific antibodies^13,28^. The secreted form of NS1 protein is found in the blood circulation at levels correlated with the severity of dengue disease^29–31^. The soluble form of NS1 contributes to enhancement of DENV production, immune evasion of DENV, induction of proinflammatory cytokine production, disruption of endothelial barrier, and vascular leakage^32–41^.

Using peptides as therapeutic agents for DENV infection has been explored previously. These peptides were designed to block active sites of viral proteins or to mimic specific regions of viral proteins as competitive inhibitors of virus entry and viral replication. Reported targets of peptide inhibitors against DENV infection include viral structural proteins C, prM, and E as well as viral NS2B/NS3 protease and NS5 methyltransferase^42–52^. In this study, we investigated DENV NS1 as a novel target of peptide inhibitors for DENV infection. A biopanning assay was performed to screen for DENV NS1-binding peptides using a phage-displayed peptide library. Identified peptides were analysed for their potential binding sites on the DENV NS1 structure and tested for their inhibitory effects on DENV production. DENV NS1-binding peptides exhibited varying degrees of inhibition of infectious virus production among different DENV serotypes. The results of this study suggest an alternative antiviral therapeutic approach against dengue by targeting the DENV NS1 protein.

## Results

### Identification of DENV NS1-binding peptides

**Figure 1:**
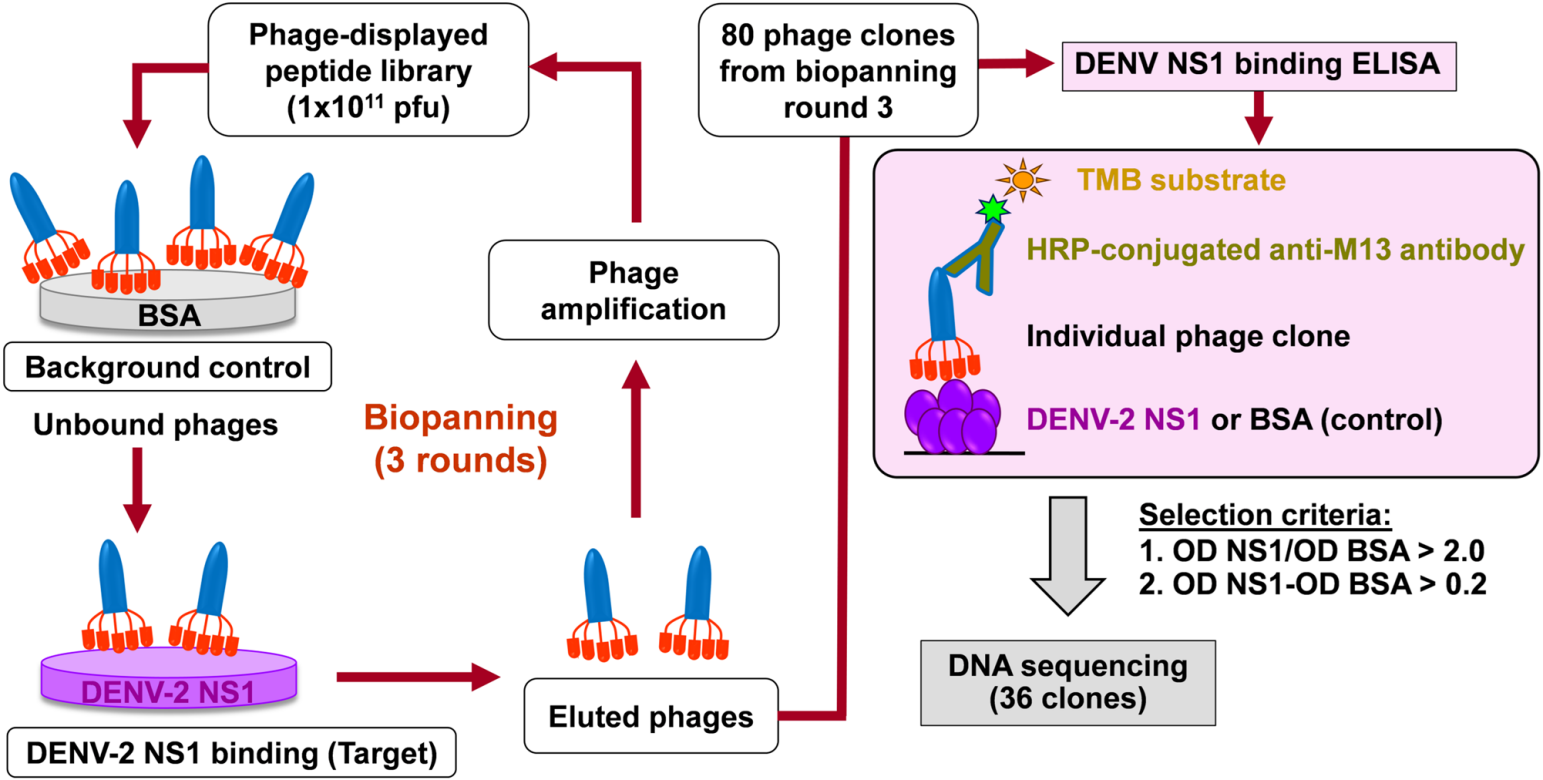
Experimental strategy for screening DENV NS1-interacting peptides by biopanning of a phage-displayed peptide library. Phages displaying 12-mer peptides (10^11^ pfu) were used for each round of biopanning, which included sequential binding of BSA (background control) and DENV NS1 (target). Eluted phages from each round were amplified in *E. coli* ER2738 and the same phage number (10^11^ pfu) was used for repeated rounds of biopanning. This process was performed for 3 rounds. Eighty clones of resulting phages from the third round of biopanning were randomly selected and verified for the binding activity of peptides with DENV NS1 by ELISA. Phage clones passing the selection criteria were subjected to DNA sequencing of a 12-mer peptide-coding region.

**Figure 2:**
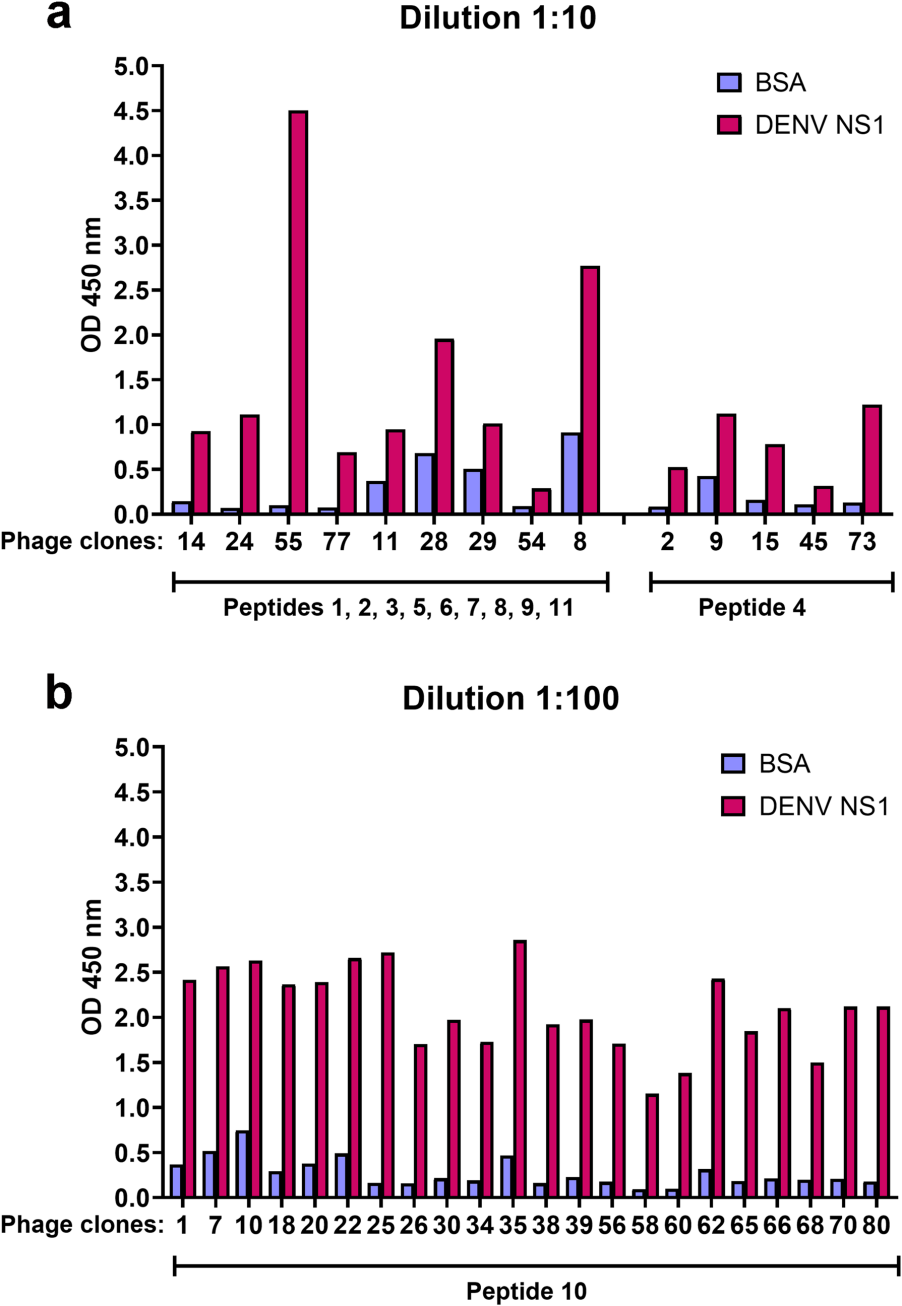
Confirmation of phage clones displaying DENV NS1-binding peptides by ELISA. Eighty phage clones were randomly selected and verified for DENV NS1 binding activity by ELISA using BSA (background control) or purified DENV-2 NS1 as an antigen. At a phage dilution of 1:10 (**a**) or 1:100 (**b**), there were 36 phage clones that yielded an OD reading ratio (DENV NS1/BSA) more than 2.0, and an OD difference between BSA and DENV NS1 more than 0.2. These clones were subjected to DNA sequencing in a peptide-coding region (9 clones containing peptides 1, 2, 3, 5, 6, 7, 8, 9, and 11; 5 clones containing peptide 4; and, 22 clones containing peptide 10).

A biopanning assay using a phage-displayed peptide library was conducted as described in the Methods section to search for DENV NS1-binding peptides. A schematic diagram illustrating the overall experimental strategy for identification of DENV NS1-binding peptides is shown in Fig. 1. Three rounds of biopanning were performed using purified NS1 from DENV-2 as the target and a fixed amount of phage input (10^11^ plaque-forming unit, pfu). Phage output in each round was determined for phage titers (Supplementary Fig. S1). The efficiency of phage recovery in biopanning rounds 2 and 3 was 29- and 350-fold, respectively, higher than in biopanning round 1 (Supplementary Fig. S1). The observed higher titer of phage output over time was indicative of successive enrichment of phage binding selectively to DENV NS1. Eighty individual phage clones were randomly selected from biopanning round 3, and the binding activities of displayed peptides towards DENV NS1 were quantitatively confirmed by enzyme-linked immunosorbent assay (ELISA). DENV-2 NS1 and BSA were used as a test antigen and a background control, respectively. All phage clones were initially diluted at 1:10 for use in this assay. Phage clones that yielded an optical density (OD) reading exceeding the maximum limit of detection (OD > 4.5) were further diluted at 1:100. Thirty-six of 80 clones exhibited OD ratios of DENV NS1-coated wells to BSA-coated wells > 2.0 and OD differences between DENV NS1-coated and BSA-coated wells > 0.2 (14 clones at a 1:10 dilution and 22 clones at a 1:100 dilution) (Fig. 2). These phage clones were then subjected to DNA sequencing analysis of nucleotide sequences coding for 12-mer displayed peptides. Nucleotide sequences and their corresponding amino acid sequences identified from 36 phage clones are presented in Supplementary Table S1. Eleven peptides were identified to effectively bind to the DENV-2 NS1 protein under the above selection criteria, including peptides 1, 2, 3, 5, 6, 7, 8, 9, and 11 from 9 different phage clones; peptide 4 from 5 phage clones; and peptide 10 from 22 phage clones (Fig. 2 and Supplementary Table S1).

### Association of identified peptides with DENV NS1 protein

**Figure 3:**
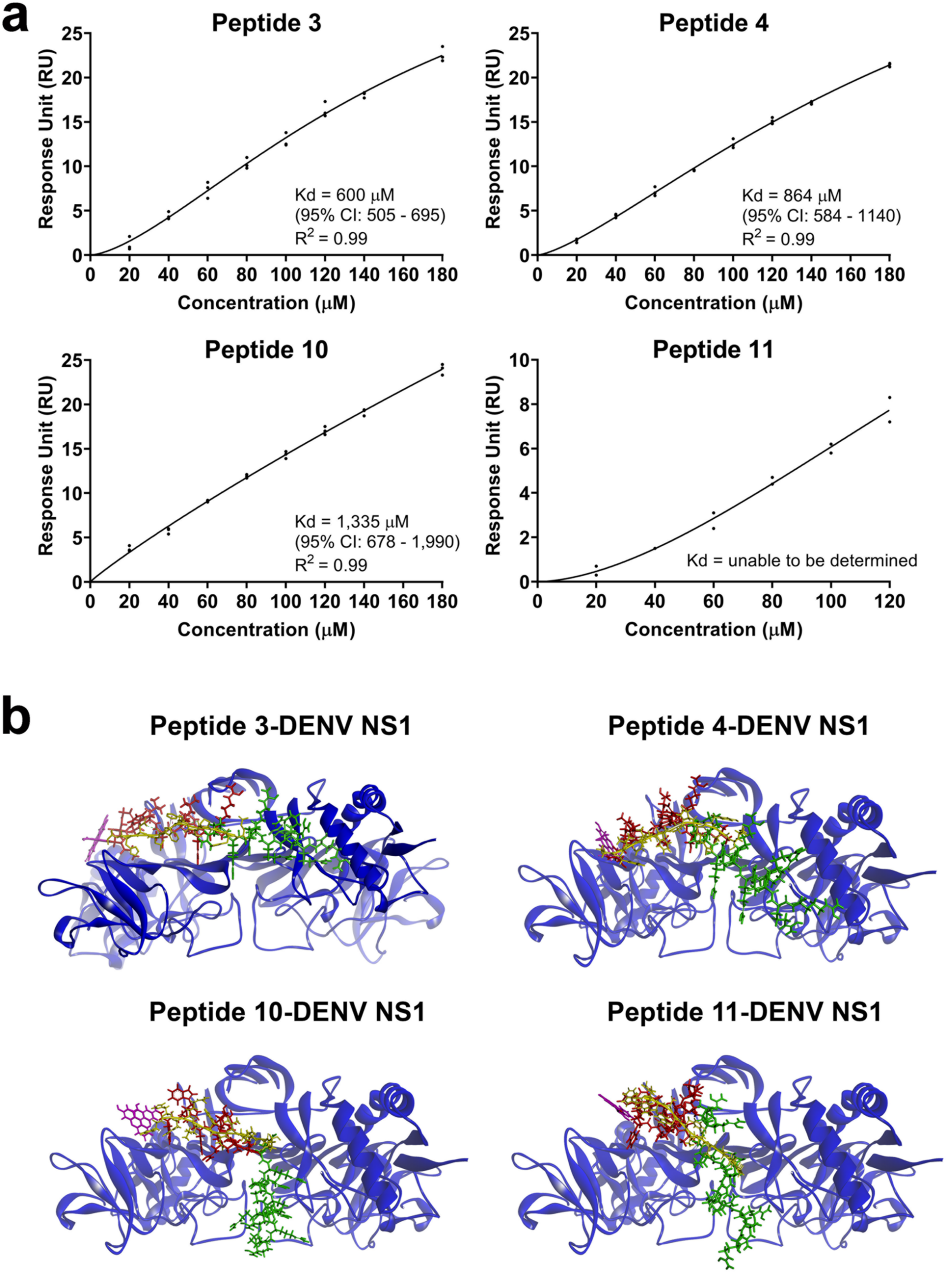
Association of identified peptides with DENV NS1. (**a**) Surface plasmon resonance assays for peptide binding to DENV NS1. Peptides 3, 4, 10 and 11 (analytes) at the indicated concentrations were analysed for their interaction with DENV NS1 (ligand) using Biacore X100. Binding responses after reference control subtraction were reported in response units (RU). Fitting kinetic curves with a saturation binding model was performed to estimate the equilibrium dissociation constant (Kd) and its 95% of confidence interval (CI) of each peptide. Results show binding responses of peptides from 2–3 independent experiments. (**b**) Molecular docking and MD simulations for binding sites of peptides with and without fluorescent and cell penetrating tags on the structure of DENV NS1. The results show binding of peptide 3, peptide 4, peptide 10, and peptide 11 with DENV NS1. Binding sites of peptide sequence (red) with the N-terminal 5-FAM (pink) and the C-terminal cell penetrating tag (green) were compared with that of the peptide sequence with no tag (yellow) on the DENV NS1 dimer (dark blue and light blue).

Molecular docking and molecular dynamics (MD) simulation were performed to predict the complex formation between the identified peptides and the DENV-NS1 protein. All 11 peptides showed differences in binding free energy values and the numbers of hydrogen and hydrophobic bonds for their interaction with the DENV NS1 protein (Table 1). Peptides 3, 4, 10, and 11 were the 4 peptides with the greatest negative values of binding free energy for complex formation, which likely indicates spontaneous DENV NS1 binding (Table 1). Further analyses of potential binding sites between the peptide-DENV NS1 complexes revealed different binding positions of all 4 peptides on the DENV NS1 protein via hydrogen and hydrophobic bond formation (Table 2). Binding sites of the 4 peptides located predominantly in the β-roll and β-ladder (amino acids 1–29 and 181–352) domains as well as, to a lesser extent, in the connector subdomains of the wing (amino acids 30–37 and 152–180) of the DENV NS1 protein (Table 2). Nevertheless, some common binding sites recognized by ≥ 2 peptides could also be observed at specific amino acid residues on the DENV NS1 structure, including Lys9, Lys14, His26, Trp28, Lys189, Arg192, Lys214, and Arg324. Further study was also carried out to determine the interaction of the 4 identified peptides with DENV NS1 protein using surface plasmon resonance assays. All 4 peptides could bind to DENV NS1 in a dose-dependent manner (Fig. 3a and Supplementary Fig. S2). Binding analyses based on a fitting model revealed the estimated values of the equilibrium dissociation constant (Kd) of peptides 3, 4, and 10 at 600 μM, 864 μM, and 1,335 μM, respectively (Fig. 3a). The Kd of peptide 11 could not be determined under the condition tested possibly due to its weaker binding affinity compared to other peptides. Responses of peptide binding, albeit to varying degrees, suggested the direct association of all 4 peptides with DENV NS1 protein.

**Table 1:** Molecular docking and MD simulations of peptide-DENV NS1 complexes.

Peptides	Amino acid sequence	Binding free energy for interaction with DENV NS1 (kcal/mol)	Number of hydrogen bonds with DENV NS1	Number of hydrophobic bonds with DENV NS1
Peptide 1	TLFSKPYPNSSR	− 7.45	8	6
Peptide 2	TPMHYPATPSPH	− 4.25	6	6
Peptide 3	QFGPVFTWLNHA	− 8.78	7	4
Peptide 4	SFVNLWTPRYSL	− 8.41	10	6
Peptide 5	TITNAPIKDLTP	− 2.63	4	5
Peptide 6	LTPHKHHKHLHA	− 6.87	13	4
Peptide 7	DPHGSLFPRTHP	− 3.55	5	4
Peptide 8	TQYPIDGDIFRR	− 5.41	3	4
Peptide 9	HLTWIPSVVRNS	− 7.24	5	4
Peptide 10	WHWRLWDVPDNP	− 9.12	6	5
Peptide 11	WHWAWYSPTARM	− 8.24	8	8

**Table 2:** Potential binding sites between DENV NS1 and identified peptides.

Peptides	Hydrogen bonds on DENV NS1	Hydrogen bonds on peptide	Hydrogen bond length (Å)	Hydrophobic bonds on DENV NS1	Hydrophobic bonds on peptide	Hydrophobic bond length (Å)
Peptide 3	Lys214	Gln1	2.41	Lys9	Pro4	4.47
	His26	Val5	2.19	Lys9	Val5	4.12
	Glu203	Trp8	2.31	Trp28	Val5	4.21
	Glu203	Trp8	2.20	Lys11	Trp8	4.42
	Glu156	Leu9	2.14			
	Lys14	Tyr10	2.36			
	Asn10	Glu12	2.32			
Peptide 4	Arg324	Ser1	2.31	Lys272	Phe2	4.18
	Arg324	Ser1	2.33	Val5	Asn4	4.23
	Glu274	Phe2	2.45	Lys9	Arg9	4.30
	Glu274	Phe2	2.98	Lys189	Trp6	4.28
	Trp28	Phe2	2.52	Arg192	Pro8	4.50
	Trp28	Val3	2.17	Lys214	Tyr10	4.25
	Arg324	Asn4	2.05			
	Trp8	Arg9	2.30			
	Trp8	Arg9	2.25			
	Asn166	Leu12	2.47			
Peptide 10	Ser7	Pro9	2.41	Lys9	Val8	4.12
	Arg192	Asp7	2.23	His26	Arg4	4.54
	Lys214	Trp6	2.04	Trp28	Arg4	4.36
	Glu326	Trp6	2.35	Arg324	Trp3	4.32
	His26	Arg4	2.26	Arg324	Trp3	4.28
	Glu326	Trp1	1.98			
Peptide 11	Gln31	Arg11	2.34	Phe277	Trp1	3.89
	Tyr32	Thr9	2.38	Phe277	Trp1	4.65
	Lys33	Arg11	1.98	Lys14	Pro8	4.85
	Phe277	Trp1	2.40	Ala205	Ala4	4.51
	Asp278	Trp1	2.28	Tyr32	Ala10	4.36
	Asp1	Ser7	2.16	Lys214	Trp1	4.16
	Glu12	Ser7	2.23	Lys9	Trp5	4.25
	Tyr32	Pro8	2.36	Lys189	Tyr6	4.21

To design DENV NS1-binding peptides that can block DENV production in target cells, we performed in silico synthesis of the selected 4 peptides in the presence of a C-terminal cell-penetrating tag and an N-terminal fluorescent tag for peptide entry and tracking in the cells. The chemical structures and predicted properties of these tag-conjugated peptides are demonstrated in Supplementary Table S2. The potential binding sites of tag-conjugated peptides on the DENV NS1 structure were analysed by molecular docking and MD simulations as described above, and then compared with those recognized by the peptides without additional tags. After conjugation with fluorescent and cell-penetrating tags, all of the 4 peptides still bound to the same grooves on the DENV NS1 structure even though there were some changes in specific amino acid positions for peptide binding (Fig. 3b, Table 2, and Supplementary Table S3). Most of the DENV NS1-binding sites (> 60%) recognized by peptides 3, 4, and 10 remained unchanged following the tag addition, whereas the binding sites of tag-conjugated peptide 11 slightly shifted to nearby positions within 1–4 amino acid residues on the DENV NS1 protein as compared to peptide 11 alone (Fig. 3b, Table 2, and Supplementary Table S3). Tag conjugation did not alter some common DENV NS1-binding sites, which could be recognized by ≥ 2 peptides, such as Lys9, His26, Trp28, Arg192, Lys214, and Arg324 (Table 2 and Supplementary Table S3). Additional amino acid positions, including Trp8, Glu203, Lys272, and Glu274, on the DENV NS1 protein were also found to be new common binding sites for ≥ 2 peptides following tag conjugation. In addition to molecular docking and MD simulations, the tag-conjugated peptides at varying concentrations showed direct interaction with DENV NS1 protein in binding ELISA (Supplementary Fig. S3). Therefore, the designed peptides with conjugated tags, which showed relatively similar DENV NS1 binding patterns to their unconjugated counterparts, were later used in this study to test inhibitory effects on DENV production.

### Inhibition of DENV NS1 binding peptides on DENV-2 production

**Figure 4:**
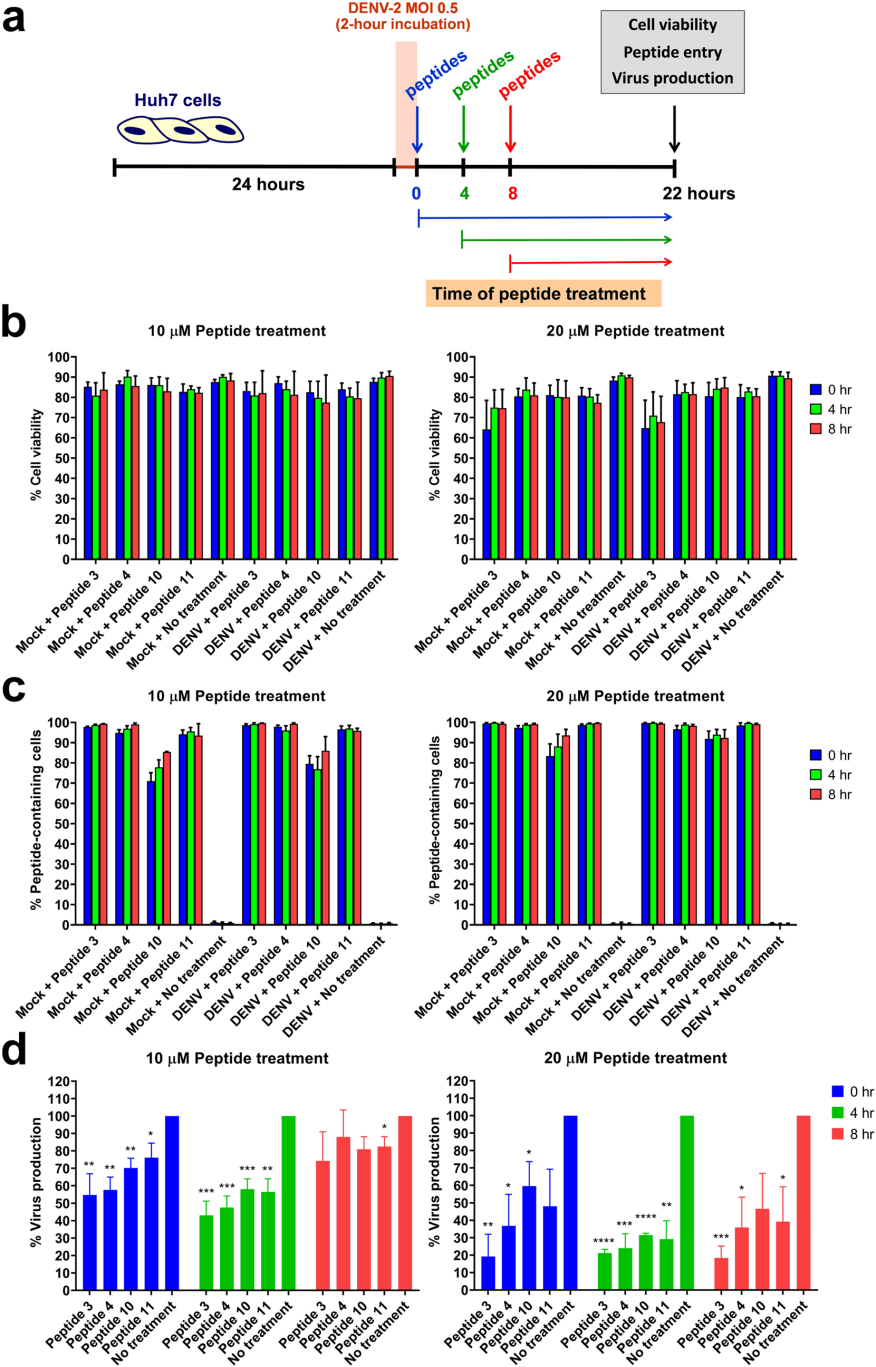
Effects of DENV NS1-binding peptide treatment on DENV-2 infection. (**a**) Huh7 cells were cultured for 24 h and then incubated with DENV-2 (strain 16681) at an MOI of 0.5 or culture medium (mock control) for 2 h. Mock and DENV-infected cells were subsequently treated with 10 µM or 20 µM of peptide 3, peptide 4, peptide 10, and peptide 11 or left untreated (no treatment) at 0, 4, and 8 h after the virus incubation period. At 24 h post-infection, cells were harvested and assessed for viability (**b**) and peptide entry (**c**) by propidium iodide staining and flow cytometry. In addition, culture supernatants collected at the same time point were determined for the production of infectious DENV by FFU assays (**d**). Results show mean + SEM of 3–4 independent experiments. DENV production was compared between peptide treatment and no treatment using unpaired *t*-test (*p < 0.05; **p < 0.01; ***p < 0.001; ****p < 0.0001).

Huh7 cells were uninfected (mock control) or infected with DENV-2 virus, then treated with 10 µM and 20 µM of peptides 3, 4, 10, and 11 conjugated with the fluorescent and cell-penetrating tags at 0, 4, and 8 h after DENV infection, and harvested at 24 h after virus exposure to determine cell viability, peptide entry, and DENV production (Fig. 4a). Our results showed that treatment with 10 µM of all 4 peptides at varying time points post-infection did not significantly affect cell viability since more than 77% of mock and DENV-infected cells were still viable following peptide treatment, as compared with 88–91% cell viability of non-treated controls (Fig. 4b, left panel). The viability of mock and DENV-infected cells remained unchanged after treatment with a higher concentration (20 µM) of peptides 4, 10, and 11 at any time point post-infection, whereas treatment with peptide 3 using the same conditions reduced cell viability to 64–75% in mock and DENV-infected cells (Fig. 4b, right panel). Regardless of the time point of peptide treatment, fluorescent signals of the conjugated peptides were detectable in mock and DENV-infected cells at 24 h post-infection by 93–100% of cells following treatment with 10 µM and 20 µM of peptides 3, 4, and 11, and by 71–86% and 83–94% of cells following treatment with 10 µM and 20 µM of peptide 10, respectively, as compared with non-treated controls (Fig. 4c, left and right panels). These findings suggested high efficiency of peptide entry into the cells in a dose-dependent manner. All 4 peptides at the 10 µM concentration decreased DENV-2 production significantly by 25–45% and 42–57% when treated at 0 and 4 h post-infection, respectively, and, to a lesser extent, by 12–26% when treated at 8 h post-infection (Fig. 4d, left panel). Increasing the concentration of the 4 peptides (20 µM) resulted in a more significant reduction in DENV-2 production by 40–81%, 69–79%, and 53–82% in DENV-infected cells when treated at 0, 4, and 8 h post-infection, respectively (Fig. 4d, right panel). Peptide 3 seemed to be the most effective at reducing DENV-2 production; however, it should be noted that treatment with peptide 3 at the 20 µM concentration not only decreased DENV-2 production, but it also caused a higher proportion of cell death compared to treatment with other peptides at the same concentration (Fig. 4b, right panel and Fig. 4d, left and right panels).

### Differential effects of DENV NS1-binding peptides on production of different DENV serotypes

**Figure 5:**
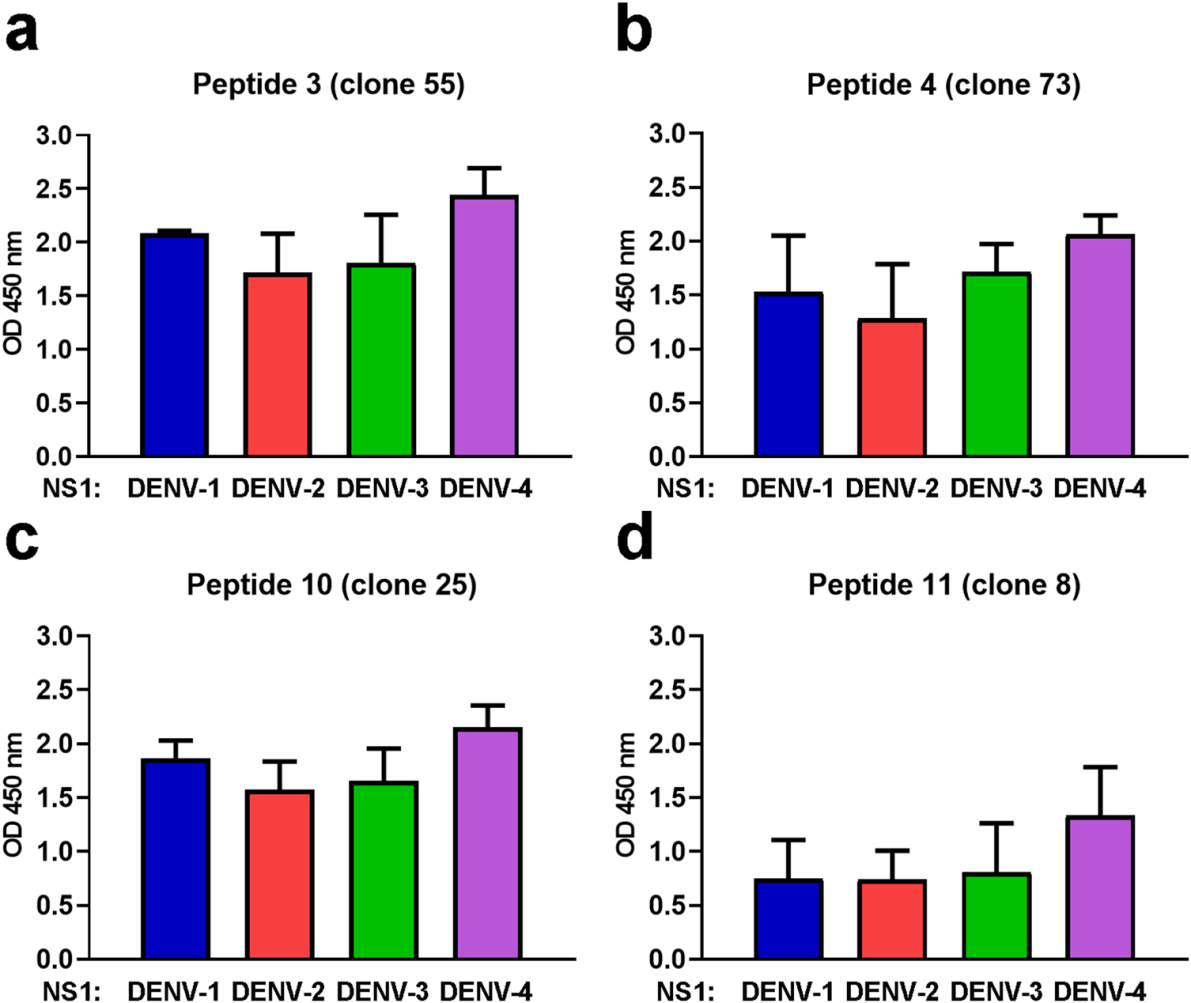
Binding of four identified peptides with DENV NS1 proteins from all four DENV serotypes. Representative phage clones displaying peptide 3, 4, 10, and 11 were cultured and concentrated for use in ELISA to determine the efficiency of peptide binding with DENV NS1 proteins from all four DENV serotypes. Results show mean + SEM of the OD reading after subtraction with background control (BSA) from 2 independent experiments.

**Figure 6:**
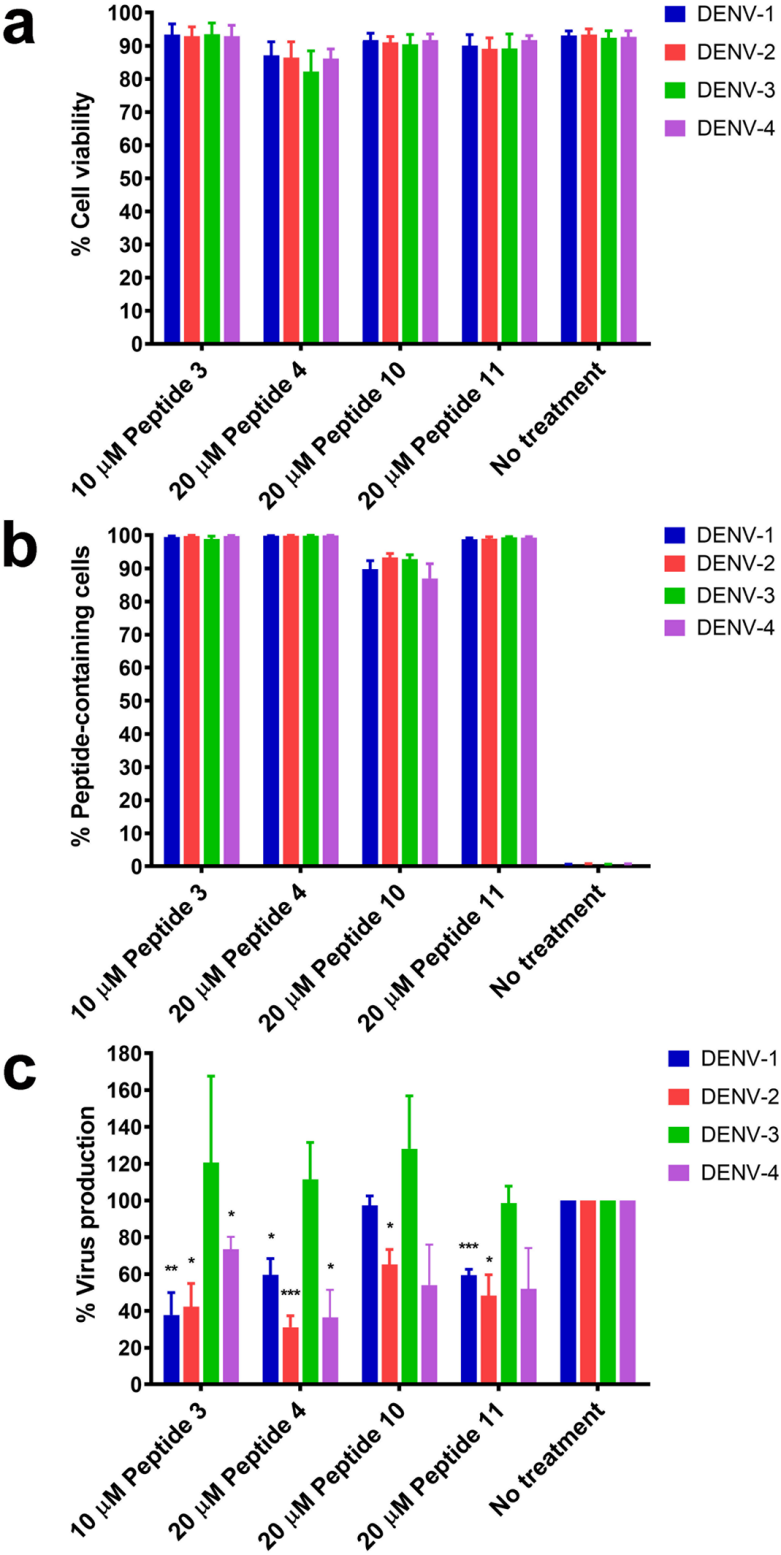
Effects of DENV NS1-binding peptide treatment on all four serotypes of DENV infection. Huh7 cells were incubated with DENV-1 (strain Hawaii), DENV-2 (strain 16681), DENV-3 (strain H87), and DENV-4 (H241) at MOIs of 2.5, 0.5, 5, and 1, respectively, for 2 h. Cells were then treated with 10 µM of peptide 3 or 20 µM of peptide 4, peptide 10, and peptide 11 at 4 h after the virus incubation period. At 24 h post-infection, cells and culture supernatants were harvested and assessed for viability (**a**), peptide entry (**b**), and production of infectious DENV (**c**) by propidium iodide staining and flow cytometry as well as FFU assays. Results show mean + SEM of 3 independent experiments. DENV production was compared between peptide treatment and no treatment using unpaired *t*-test (*p < 0.05; **p < 0.01; ***p < 0.001).

To determine whether peptides 3, 4, 10, and 11 can bind to NS1 proteins from all four DENV serotypes, representative phage clones displaying specific peptides were used in NS1-binding ELISA with purified NS1 proteins from DENV serotypes 1, 2, 3, and 4 as target antigens. The results showed that peptides 3, 4, 10, and 11 expressed by the corresponding phage clones had comparable efficiencies to bind DENV NS1 proteins of all four virus serotypes (Fig. 5). Therefore, further investigation was conducted to examine the inhibitory effect of these NS1-binding peptides against DENV production for the four serotypes. Huh7 cells were infected with different serotypes of DENV, as described in the Methods section, and subsequently treated with 10 µM of peptide 3 and 20 µM of peptides 4, 10, or 11 at 4 h post-infection. DENV-infected cell cultures were assessed for cell viability, peptide entry, and DENV production at 24 h after infection. The infection conditions used for all four DENV serotypes yielded similar percentages of cells expressing DENV NS1 antigen, an indicative marker for DENV infection, by approximately 56%-67% as evidenced by immunofluorescence staining (Supplementary Fig. S4). Regardless of the serotype of DENV infection, virus-infected cells exhibited more than 82% viability under all conditions of peptide treatment as compared with non-treated controls (Fig. 6a). Peptide treatment resulted in the detection of its fluorescent tag signal in virtually all of the DENV-infected cells following treatment with peptides 3, 4, and 11 and, to a lesser extent, in approximately 87–93% of cells following treatment with peptide 10 at 24 h post-infection (Fig. 6b). Under the conditions tested, peptides 3 and 4 could significantly inhibit the production of DENV-1, -2, and -4 from virus-infected cell cultures to varying degrees, whereas peptide 10 showed significant inhibitory effects only on DENV-2 production by 35% reduction (Fig. 6c). Peptide 11 resulted in significant decreases of DENV-1, and -2 production by 41–52% (Fig. 6c). In addition, treatment with either peptide 10 or peptide 11 tended to reduce DENV-4 production by approximately 50%, although there was no statistically significant difference (Fig. 6c). Higher percentage reduction of DENV production was observed predominantly for DENV-1 (62%) and DENV-2 (58%) following peptide 3 treatment, and for DENV-2 (69%) and DENV-4 (64%) following peptide 4 treatment (Fig. 6c). Interestingly, no peptide treatment affected DENV-3 production when compared with non-treated control (Fig. 6c). Consistent with DENV production, treatment with peptides 3, 4, 10, and 11 resulted in a reduction of intracellular DENV NS1 expression after infection with DENV-1, -2, and -4, but not DENV-3 (Supplementary Fig. S5). Taken together, these findings suggest potential differences in peptide accessibility to specific binding sites on different serotypes of DENV NS1.

## Discussion

Development of antiviral agents against DENV infection involves different strategies to inhibit the life cycle of DENV in host cells. The use of peptide inhibitors that influence the function of viral proteins has drawn attention as an alternative approach for potential treatment of DENV infection. This study used the DENV NS1 protein, which is an important viral non-structural protein required for survival of the infectious virus in the host, as a target for screening peptide inhibitors against DENV infection from a phage-displayed peptide library. A set of identified peptides were confirmed for their binding activities with DENV NS1 by ELISA and characterized for DENV NS1 interaction by molecular docking, MD simulations, and surface plasmon resonance assays. Four identified peptides were highlighted for their specific binding sites on the NS1 structure, and for their differential inhibitory effects on all four serotypes of DENV production in virus-infected cell cultures.

Several studies in peptide inhibitors against DENV infection previously concentrated on different viral targets, including viral enzymes and all three structural proteins of DENV. One strategy for the development of peptide inhibitors is to design peptides mimicking amino acid sequences of viral proteins that may interfere with the interactions between viral proteins and host components essential for different steps in the virus life cycle. A peptide corresponding to a conserved N-terminal motif of flavivirus C protein at amino acid residues 14–23 was found to inhibit binding of DENV C with lipid droplets and very low-density lipoproteins, which are required for the formation of virus particles^53,54^. Peptides containing the amino acid residues 25–42, 98–109, and 340–354 of domains I, II, and III of DENV E protein reduced the infection of all four DENV serotypes by binding with the E protein on the surface of virion and by blocking virus entry^55^. Another study using peptide sequences corresponding to the stem domain of DENV E (amino acids 412–444 and 419–447) also showed inhibition of DENV infectivity via peptide binding with viral particles that results in disruption of viral membrane and release of viral genome as well as prevention of E trimer fusion with the endosomal membrane^43,56–58^. In addition to the DENV E protein, a peptide that mimics the conserved ectodomain region of the DENV M protein inhibited all four serotypes of DENV in different target cell lines possibly due to interference of DENV M-E protein interaction^46^.

The other strategy for development of antiviral peptides is to search for peptide inhibitors that can bind to specific targets and interfere with their functions. Cyclopentapeptides and cyclic peptides have been screened for binding activities with S-adenosyl methionine binding site and RNA-cap site of DENV NS5 methyltransferase by molecular docking and MD simulation; however, their potential inhibition against DENV infection has not been tested in virus-infected cells^44,59^. Cyclic peptides that mimic the substrates of the DENV NS2B-NS3 protein exhibited inhibitory activities on viral protease and anti-viral activities against DENV-2^60^. Cationic peptides from natural sources, such as plectasin, which is a fungus-derived defensin, as well as latarcin-1 and defensin-like An1a peptides from spider venom targeted NS2B-NS3 protease of DENV-2, resulting in the inhibition of viral protease activity and DENV-2 replication^61–63^. Di- and tripeptides with β-lactam moiety could serve as electrophilic warheads in the reduction of DENV NS2B-NS3 protease activity and infectious virus production^64^. In addition, di- and tripeptides targeting the hydrophobic pocket of the DENV E protein, which involves conformational changes during the step of virus fusion, could effectively inhibit DENV-2 infection in Vero cells^65^. Another study used a murine brain cDNA phage display library for screening specific peptides against the E protein of West Nile virus (WNV), a member of flavivirus, and showed in vitro and in vivo effects of peptide inhibition on WNV infection^66^.

In this study, we proposed DENV NS1 as a novel target for inhibition of DENV infection by identifying specific peptide inhibitors from a Ph.D.-12 phage display peptide library. Utilization of the same peptide library proved successful for identifying a set of peptides binding to purified DENV-2 and E protein (amino acid residues 1–400 and domain III) of Japanese encephalitis virus (JEV), a related flavivirus, and to demonstrate peptide inhibition of DENV-2 infection in Vero cells and JEV infection in BHK-21 cells during the step of virus entry as well as in a mouse model with lethal JEV challenge^67–69^. Our results of biopanning assays revealed that the specificity of DENV NS1 binding by phage clones increased over time as evidenced by the efficiency of phage recovery after each round of selection, and that 36 out of 80 phage clones from the third round of biopanning satisfied our selection criteria based on DENV NS1 binding activities and displayed 11 different sequences of 12-mer peptides. Further study in the interactions of the 11 identified peptides with the DENV NS1 structure by molecular docking and MD simulations demonstrated peptides 3, 4, 10, and 11 to be the 4 peptides most likely to bind with DENV NS1 based on their high negative values of binding free energy for complex formation (Table 1). Direct association of these 4 peptides with DENV NS1 protein was evidenced by the surface plasmon resonance assays with the estimated Kd values at a range of micromolar to millimolar levels (Fig. 3a).

In DENV-infected cells, intracellular DENV NS1 protein localizes in the luminal side of the endoplasmic reticulum, is required for the formation of membrane organelles derived from ER invagination as the site of viral RNA replication (also known as vesicle packets), and plays an important role in the production of infectious virus particles^19,20,70^. To target the DENV NS1 protein in infected cells, the 4 selected peptides were designed to have accessibility and traceability into the cells via the addition of an N-terminal fluorescent tag and a C-terminal cell penetrating tag, as previously demonstrated^71^. Since the DENV NS1 protein is not a structural component of the virus particle and must be synthesized inside virus-infected cells, it was expected that specific inhibition against the NS1 protein could take place after the step of virus entry, most likely following viral RNA translation. Previous studies using DENV-2 reporter replicon suggest that DENV RNA translation likely occurs in the first 4–8 h post-infection as revealed by kinetic assays for luciferase activities of the reporter virus following RNA transfection^72–74^. Therefore, time-of-addition assays for peptide treatment were performed in the present study at 0 h (prior to the beginning of viral RNA translation), 4 h (the first round of actively ongoing viral RNA translation), and 8 h (after the first round of viral RNA translation and ongoing viral RNA replication) following DENV infection for target inhibition of the DENV NS1 protein post-virus entry. All 4 peptides could efficiently enter the cells, and no significant cytotoxicity was observed after treatment at any time points. However, it should be noted that peptide 10 was detectable in cells to a lesser degree, and peptide 3 at the higher concentration (20 µM) seemed to yield a greater degree of cell death at 24 h after mock or DENV infection when compared with other peptides (Fig. 4b,c). The different degrees of peptide detection in cells may reflect differences in the efficiency of peptide entry and/or peptide stability in cells. Treatment with any of the 4 peptides resulted in a reduction of DENV-2 production in a time- and dose-dependent manner (Fig. 4d). At the 10 µM concentration, all 4 peptides showed better efficacy for decreasing DENV-2 production when applied before or during actively ongoing viral RNA translation (0- and 4-h time points), as compared with those applied after the first round of viral RNA translation (8-h time point) (Fig. 4d, left panel). Nevertheless, increasing the concentration of peptides to 20 µM could enhance the inhibitory effects on DENV-2 production by peptide treatment at all time points (Fig. 4d, right panel).

Bases on our findings, treatment with 20 µM peptides (except for peptide 3 with 10 µM) at 4 h post-infection when viral RNA translation is actively ongoing was applied to Huh7 cell cultures infected with all four serotypes of DENV to further test whether the peptide inhibition against DENV production is serotype-dependent. Peptide treatment under these conditions significantly reduced the infectious virus production of DENV-1, DENV 2, and DENV-4, but not DENV-3 (Fig. 6). Inability of peptides to inhibit DENV-3 production was unlikely due to different degrees of virus infection among different serotypes because comparable percentages of DENV-infected cells were observed under the infecting conditions used for individual virus serotypes (Supplementary Fig. S4). Although NS1 ELISA using peptide-displayed representative phage clones demonstrated the capability of peptides 3, 4, 10, and 11 to bind to all four serotypes of DENV NS1 (Fig. 5), none of these peptides revealed inhibitory efficacy against DENV-3 production in virus-infected cultures (Fig. 6). In the ELISA system, DENV NS1 proteins were coated directly onto plates as the target antigens to be recognized by the peptides from the phage clones. Thus, it is possible that the structural conformation of the DENV-3 NS1 protein when attached to the solid surface is present in a form that favors peptide recognition as compared to the form of the DENV-3 NS1 protein in virus-infected cells, which might be less amenable to peptide binding. A possibility that the peptide binding sites are occluded by DENV NS1-interacting host proteins in virus-infected cells, which may act differently among different DENV serotypes, could not also be ruled out. Our analyses by molecular docking and MD simulations demonstrated potential binding sites of all 4 peptides on the structure of DENV-2 NS1 that are predominantly located in the β-roll and β-ladder domains and share similarity of several amino acid residues with other serotypes of DENV NS1. Our observation that all 4 peptides could bind mostly to the conserved amino acid residues of four serotypes on the DENV NS1 protein, but not inhibit DENV-3 production, suggests potential differences in the conformational structure of DENV NS1 among the different virus serotypes, which may determine differential ligand recognition.

The majority of the peptide binding sites seemed to be unchanged following the addition of fluorescent tag and cell penetrating tag to the peptides, except for those of peptide 11, which demonstrated a slight shift to nearby positions within 1–4 amino acid residues on the DENV NS1 protein as compared to peptide 11 alone (Table 2 and Supplementary Table S3). Peptides 3, 4, 10, and 11 showed different binding positions on the DENV NS1 structure, which might determine the fate of peptide inhibition against DENV production; however, some common binding sites could still be observed. The DENV NS1 Lys9 residue is putatively recognized by all 4 peptides and localized in close proximity to Trp8, which is required for DENV RNA replication, but not association with NS4A-2K-4B precursor^19,20^. His26 and Trp28 putatively interact with ≥ 2 peptides and are located in the β-roll domain (amino acid residues 1–29), which is likely involved in NS1 dimerization and association with ER membrane and other viral transmembrane proteins^18,19^. A hydrophobic pocket involved in the monomer–monomer interaction of the DENV NS1 protein was also proposed in a recent study as a target for drug design to disrupt the structure of DENV NS1, particularly in the region consisting of Phe20, Ile21, and Trp201^75^. Furthermore, we found Arg192 to be recognized by peptides 4 and 10 in the β-ladder domain close to Lys189, which is a critical amino acid residue for efficient DENV RNA replication^19,20^. The present study also identified Lys189 as a unique binding site for peptides 4 and 11; however, the recognition of this binding position appeared to be altered following the tag addition (Table 2 and Supplementary Table S3). The unique binding site for peptide 3 at Glu156 was found to reside in the β-ladder domain at a position adjacent to Tyr158 that is essential for RNA replication of DENV^19,20^. Therefore, specific binding sites on the DENV NS1 structure that are recognized by these peptides may be important factors for determining the efficiency of peptide inhibition against DENV infection.

Several approaches have been developed to search for antiviral peptides to control DENV infection. However, for the first time, the present study proposes the multifunctional DENV NS1 protein as a novel target for inhibition of DENV infection by specific peptide inhibitors. Treatment with DENV NS1-binding peptides reduced infectious virus production even after the step of virus entry into the cells; however, the detailed mechanisms of peptide inhibition require further investigation and elucidation. Our identification of specific peptide binding sites on the DENV NS1 structures and differential peptide inhibition of DENV production among different virus serotypes may guide the future development of a DENV NS1-based design for specific inhibitors or drugs against DENV infection.

## Methods

### Cell line, virus, antibodies and peptides

Hepatocellular carcinoma Huh7 cells were cultured in 10% FBS-DMEM (Gibco; Invitrogen, Carlsbad, CA, USA) containing 0.1 mM non-essential amino acid, 2 mM l-glutamine, 36 µg/ml penicillin G, and 60 µg/ml streptomycin at 37 °C with 5% CO_2_ and a humidified atmosphere. Dengue virus serotypes (DENV-1 strain Hawaii, DENV-2 strain 16681, DENV-3 strain H87, and DENV-4 strain H241) were propagated in mosquito C6/36 cells. A mouse monoclonal antibody specific for DENV NS1 (clones NS1-3F) was produced in our laboratory^76^. Alexa Fluor 488-conjugated goat anti-mouse IgG antibody and Cy3-conjugated goat anti-mouse IgG antibody were purchased from Invitrogen and Jackson ImmunoResearch (West Grove, PA, USA), respectively. Peptide 3 (QFGPVFTWLNHA), peptide 4 (SFVNLWTPRYSL), peptide 10 (WHWRLWDVPDNP), and peptide 11 (WHWAWYSPTARM) that contained additional N-terminal 5-carboxyfluorescein (5-FAM) and C-terminal cell-penetrating peptide tag (RRRGRRRRRRRR) were synthesized at GenScript (Piscataway, NJ, USA) with purity of 97.9%, 96.4%, 98.5%, and 96.9%, respectively, and removal of standard trifluoroacetic acid.

### Biopanning of phages displaying DENV NS1-binding peptides

The Ph.D.-12 phage display peptide library (New England Biolabs, Ipswich, MA, USA), which contains random 12-mer peptides fused to a minor coat protein (pIII) of M13 phage, was used to screen peptides binding to DENV NS1. Briefly, a Nunc Maxisorp 96-well plate (Nunc, Roskilde, Denmark) was coated with 1 µg of purified NS1 protein from DENV-2-infected Vero cell culture or bovine serum albumin (BSA, background control) at 4 °C overnight. The protein-coated wells were washed 5 times with 200 µl of TBS buffer containing 0.1% Tween 20 (TBST, washing buffer) and incubated with 200 µl of 3% BSA in TBS at room temperature (RT) for 2 h. After 5 washes, the phage-displayed peptide library (10^11^ pfu) was incubated in the BSA-coated wells at RT for 1 h to deplete BSA-binding phages. Unbound phages from the BSA-coated wells were transferred to the DENV NS1-coated wells and incubated at RT for 1 h. The DENV NS1-coated wells were then thoroughly washed to remove unbound phages. DENV NS1-binding phages were eluted from the wells by incubating with glycine elution buffer (0.2 M glycine–HCl pH 2.2 and 1 mg/ml BSA) at RT for 20 min, followed by neutralization with 1 M Tris–HCl pH 9.1. The eluted phages were amplified in *E. coli* ER2738 cultures and titrated according to the manufacturer’s protocol with minor modifications. The amplified phages (10^11^ pfu) from the first round were used for the next round of biopanning, and a total of 3 rounds of biopanning were performed. The number of phages obtained from each round was titrated to evaluate the efficiency of phage recovery.

### Confirmation of phage clones for DENV NS1 binding

Eighty randomly selected phage clones were cultured in *E. coli* ER2738, and the resulting phages were precipitated with 20% PEG 8,000 in 2.5 M NaCl and resuspended in TBS according to the manufacturer’s protocol (New England Biolabs). DENV NS1 binding activity of peptides from each phage clone was verified by enzyme-linked immunosorbent assay (ELISA). A Nunc Maxisorp 96-well plate was coated with 150 ng of purified DENV-2 NS1 from virus-infected Vero cell culture or BSA at 4 °C overnight. Remaining binding sites on the wells were blocked with 3% BSA in TBS for 2 h at RT. Individual phage clones at a 1:10 or 1:100 dilution were incubated in the DENV NS1- and BSA-coated wells for 1 h at RT. After 5 washes with TBST, the wells were incubated with HRP-conjugated mouse anti-phage M13 antibody at a 1:2,500 dilution (GE Healthcare, Chicago, IL, USA) for 1 h at RT in the dark. The wells were washed 5 times and incubated with 3, 3′, 5, 5′-tetramethylbenzidine chromogenic substrate (1-Step Ultra TMB-ELISA Substrate; Thermo Fisher Scientific, Waltham, MA, USA) for 5 min at RT. The enzymatic reaction was stopped by the addition of 2 N H_2_SO_4_. Optical density (OD) was measured at 450 nm (measurement wavelength) and 620 nm (reference wavelength) using a Synergy H1 multi-mode microplate reader (Biotek Instruments, Winooski, VT, USA). Phage clones, which provide > 2.0-fold changes of OD reading in DENV NS1-coated wells to OD reading in BSA-coated wells, and > 0.2 OD difference between OD readings in DENV NS1-and BSA-coated wells, were selected for DNA sequencing (Macrogen, Seoul, South Korea) of 12-mer inserted peptides. The resulting nucleotide sequences were converted into amino acid sequences using EMBOSS Transeq (https://www.ebi.ac.uk/Tools/st/emboss_transeq). To test the binding activity of selected phage clones with NS1 protein from all four DENV serotypes, ELISA was performed as described above using purified NS1 proteins (150 ng) from Vero cell cultures infected with DENV-1, -2, -3, and -4 as target antigens.

### In silico prediction of peptide binding sites on the DENV NS1 structure

Eleven DENV NS1-binding peptide sequences were processed using Prime Module Schrödinger’s Maestro molecular modeling suite^77^. Molecular docking of these peptides was performed on the DENV-2 NS1 protein structure (PDB ID: 4O6B) from the Protein Data Bank (https://www.rcsb.org). Structural and sequence analyses of the DENV NS1 protein, as well as preparation of the ligand-target structures, were performed using Biovia Discovery Studio 2019 software^78^. Computational prediction of DENV NS1 and peptide binding was conducted using High Ambiguity Driven protein–protein docking (HADDOCK)^79,80^. Binding free energy of 2,000 poses of protein-peptide docking was calculated using the Molecular Mechanics Poisson-Boltzmann Surface Area approach (MM-PBSA)^81^, and poses with the most negative values of binding free energy were selected for further analyses of potential binding sites between peptides and DENV NS1. Nanoscale Molecular Dynamics (NAMD) 2.12 (Beckman Institute, Urbana, IL, USA)^82^ with CHARMM27 force field^83^ was used to perform the molecular dynamics (MD) simulations of peptide-DENV NS1 complex structures according to a previously described condition^25^. Results from the MD simulations were analysed for stability of the peptide-DENV NS1 complexes using Visual Molecular Dynamics (VMD) 1.9.3 (Beckman Institute)^84^. Potential binding sites of the peptide-DENV NS1 complexes were analysed by Biovia Discovery Studio 2019 software^78^.

The 4 DENV NS1-binding peptides with the lowest binding free energy were selected for computational conjugation with a fluorescent tag (5-carboxyfluorescein, 5-FAM) and a cell penetrating tag (RRR-G-R8) at the N- and C-terminus, respectively, using Prime Module Schrodinger’s Maestro molecular modeling suite. Molecular docking, MD simulations, and determination of potential binding sites of the tag-conjugated peptide and DENV NS1 complexes were performed as described above using HADDOCK, NAMD 2.12 with CHARMM27 force field, and Biovia Discovery Studio 2019 software, respectively.

### Surface plasmon resonance analysis for peptide and DENV NS1 binding

A protein G sensor chip (GE Healthcare, Uppsala, Sweden) was immobilized with 25 μg/ml of an anti-DENV E antibody (irrelevant control) and an anti-DENV NS1 antibody on flow cell 1 (reference cell) and flow cell 2 (test), respectively, at a flow rate of 5 μl/minute for 60 s twice using Biacore X100 (GE Healthcare). Purified DENV-2 NS1 protein at a concentration of 250 nM (ligand) was flowed through both flow cells at a flow rate of 5 μl/min for 30 s followed by 300 s of washing, and this process was repeated 6 times. Peptides without tag conjugation (analytes) or diluent controls were injected into the flow cells at varying concentrations with a flow rate of 10 μl/minute and contact time/ dissociation time of 30 s/ 60 s for peptides 3, 4, and 10, and 100 s/ 200 s for peptide 11. The flow cell temperature was 25 °C. Running buffers and peptide diluents consisted of 10 mM Tris–HCl pH 7.5, 150 mM NaCl and 3% dimethyl sulfoxide for peptides 3, 4 and 10, and 10 mM HEPES, 150 mM NaCl and 0.005% Tween 20 for peptide 11. Binding responses of flow cell 2 after reference subtraction were reported in response units (RU) using Biacore X100 Evaluation Software version 2.0.1. Analysis of binding responses over time was performed using GraphPad Prism version 8.3.1 (GraphPad Software, San Diego, CA, USA). The equilibrium dissociation constant (Kd) of peptides were calculated according to a previously described method^85^.

### Effects of DENV NS1-binding peptides on DENV production

Huh7 cells (2.5 × 10^4^ cells) in 96-well plates were incubated with DENV-2 at a multiplicity of infection (MOI) of 0.5 or culture medium (mock control) for 2 h. Mock and DENV-infected cells were subsequently washed 3 times with PBS and treated with different concentrations of peptides or a diluent control at 0, 4, and 8 h after the virus incubation period. At 24 h post-infection, cells and culture supernatants were harvested and assessed for cell viability, peptide entry, and production of infectious virus by propidium iodide staining and flow cytometry as well as by focus forming unit (FFU) assay^86^. To test the inhibitory effects of peptides on all four serotypes of DENV, Huh7 cells were infected with DENV-1, DENV-2, DENV-3, or DENV-4 at MOIs of 2.5, 0.5, 5, or 1, respectively, and treated with peptides or a diluent control at 4 h post-infection. Cells and supernatants were subsequently harvested at 24 h post-infection and processed as described above. To determine the percentage of DENV-infected cells, Huh7 cells infected with each of the four DENV serotypes were subjected to immunofluorescence staining with an anti-NS1 antibody and flow cytometry^25^.

### Statistical analysis

Unpaired *t*-test was performed using GraphPad Prism version 8.3.1 to analyze differences in cell viability and DENV production between each peptide-treated group and non-treated group. *P*-values < 0.05 represent statistical significance.

## Supplementary information

Supplementary file1.

## Data Availability

All data generated or analysed during this study are included in this article and its supplementary information files.
