# Supplementary material for: Peptides targeting dengue viral nonstructural protein 1 inhibit dengue virus production
Source: Sci Rep. 2020 Jul 31;10:12933. doi: 10.1038/s41598-020-69515-9 (PMC7395749; doi:10.1038/s41598-020-69515-9)
Supplement: Supplementary file 1 — Supplementary file1. [file 41598_2020_69515_MOESM1_ESM.pdf]

Peptides targeting dengue viral nonstructural protein 1 inhibit dengue virus production

Pucharee Songprakhon[1], Thanawat Thaingtamtanha[2,10], Thawornchai Limjindaporn[3], Chunya Puttikhunt[4,5,6], Chatchawan Srisawat[7], Prasit Luangaram[4,5,6], Thanyaporn Dechtawewat[1], Chairat Uthaipibull[8], Sissades Thongsima[9], Pa-thai Yenchitsomanus[1], Prida Malasit[4,5,6], Sansanee Noisakran[4,5,6,*]

[1]*Division of Molecular Medicine, Research Department, Faculty of Medicine Siriraj Hospital, Mahidol University, Bangkok, Thailand*

[2]*Faculty of Pharmacy, Rangsit University, Pathum Thani, Thailand*

[3]*Department of Anatomy, Faculty of Medicine Siriraj Hospital, Mahidol University, Bangkok, Thailand*

[4]*Molecular Biology of Dengue and Flaviviruses Research Team, Medical Molecular Biotechnology Research Group, National Center for Genetic Engineering and Biotechnology, National Science and Technology Development Agency, Bangkok, Thailand*

[5]*Division of Dengue Hemorrhagic Fever Research, Research Department, Faculty of Medicine Siriraj Hospital, Mahidol University, Bangkok, Thailand*

[6]*Siriraj Center of Research Excellence in Dengue and Emerging Pathogens, Faculty of Medicine Siriraj Hospital, Mahidol University, Bangkok, Thailand*

[7]*Department of Biochemistry, Faculty of Medicine Siriraj Hospital, Mahidol University, Bangkok, Thailand*

[8]*Protein-Ligand Engineering and Molecular Biology Research Team, Medical Molecular Biotechnology Research Group, National Center for Genetic Engineering and Biotechnology, National Science and Technology Development Agency, Pathum Thani, Thailand*

[9]*National Biobank of Thailand, National Science and Technology Development Agency, Pathum Thani, Thailand*

[10]*Present Address: Department of Chemistry and Biology, University of Siegen, Siegen, Germany*

***Corresponding author:** Dr. Sansanee Noisakran, Molecular Biology of Dengue and Flaviviruses Research Team, Medical Molecular Biotechnology Research Group, National Center for Genetic Engineering and Biotechnology, National Science and Technology Development Agency, Bangkok 10700, Thailand; Phone/Fax: (+66) 2-418-4793; E-mail: sansanee@biotec.or.th or snoisakran@yahoo.com

Keywords: Dengue virus NS1, peptide, phage-displayed library, binding site, dengue virus production

a

Round of biopanning	Titer of recovered phages (pfu)	Fold increase of phage recovery efficiency
1	1.80E+05	1
2	5.30E+06	29
3	6.30E+07	350

b

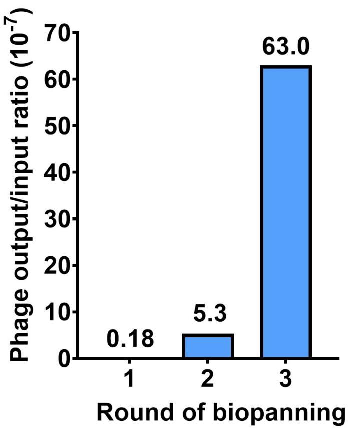

Supplementary Figure S1. Increased recovery efficiency of DENV NS1-interacting phages following each round of biopanning. Eluted phages from each round of biopanning (phage output) were titrated, and the efficiency of phage recovery was determined by comparison of phage titers in rounds 2 and 3 with round 1 of biopanning (a). The titers of phage output in each round were compared with that of phage input (10^{11} pfu), and the output/input ratio represented the efficiency of phage recovery following biopanning (b).

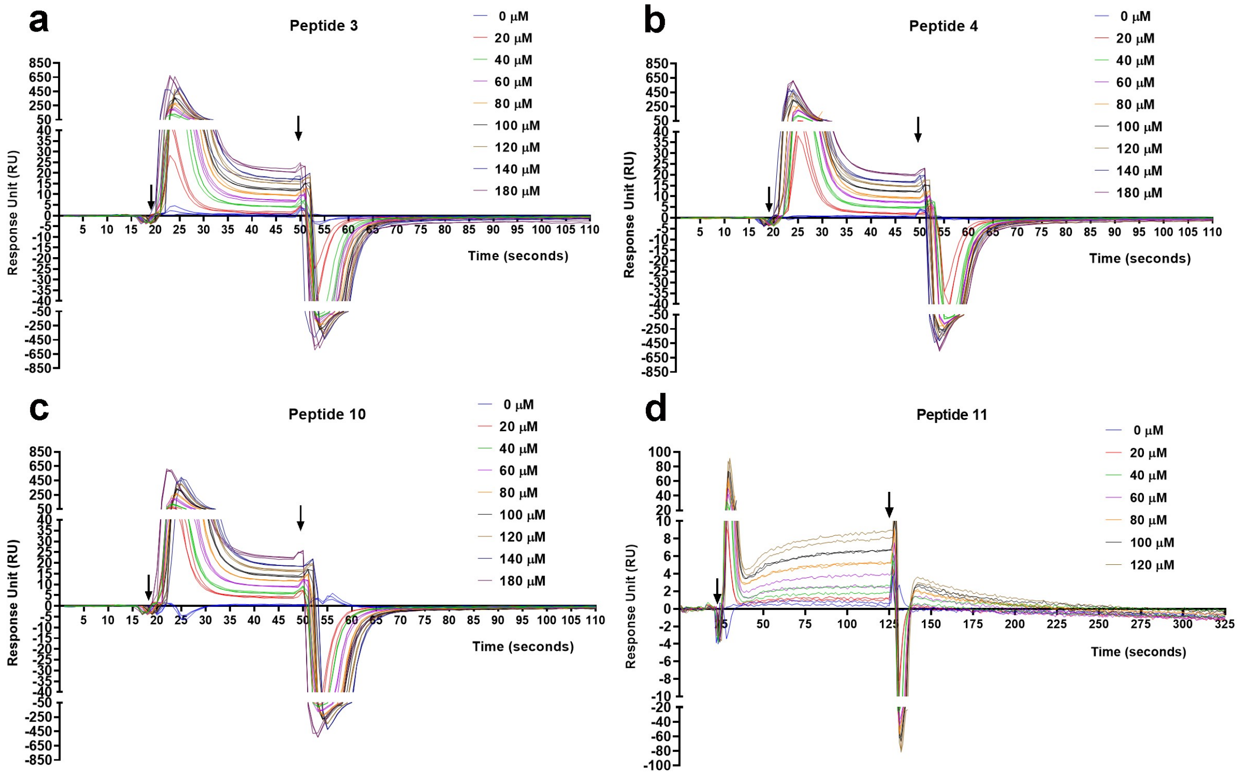

Supplementary Figure S2. Surface plasmon resonance sensorgrams of peptide binding to DENV NS1. Purified DENV-2 NS1 protein (ligand) was flowed into Biacore X100 using a protein G sensor chip immobilized with an anti-DENV E antibody (irrelevant control) and an anti-DENV NS1 antibody on flow cell 1 (reference cell) and flow cell 2 (test), respectively. Peptides (analytes) at the indicated concentrations were injected into both flow cells and binding responses of flow cell 2 after reference subtraction were reported in response units (RU) during 30 seconds of a contact time and 60 seconds of a dissociation time for peptides 3 (a), peptide 4 (b) and peptide 10 (c), or 100 seconds of a contact time and 200 seconds of a dissociation time for peptide 11 (d). Arrows represent the beginning and the end of analyte injection. Results show responses of peptide binding from 2-3 independent experiments.

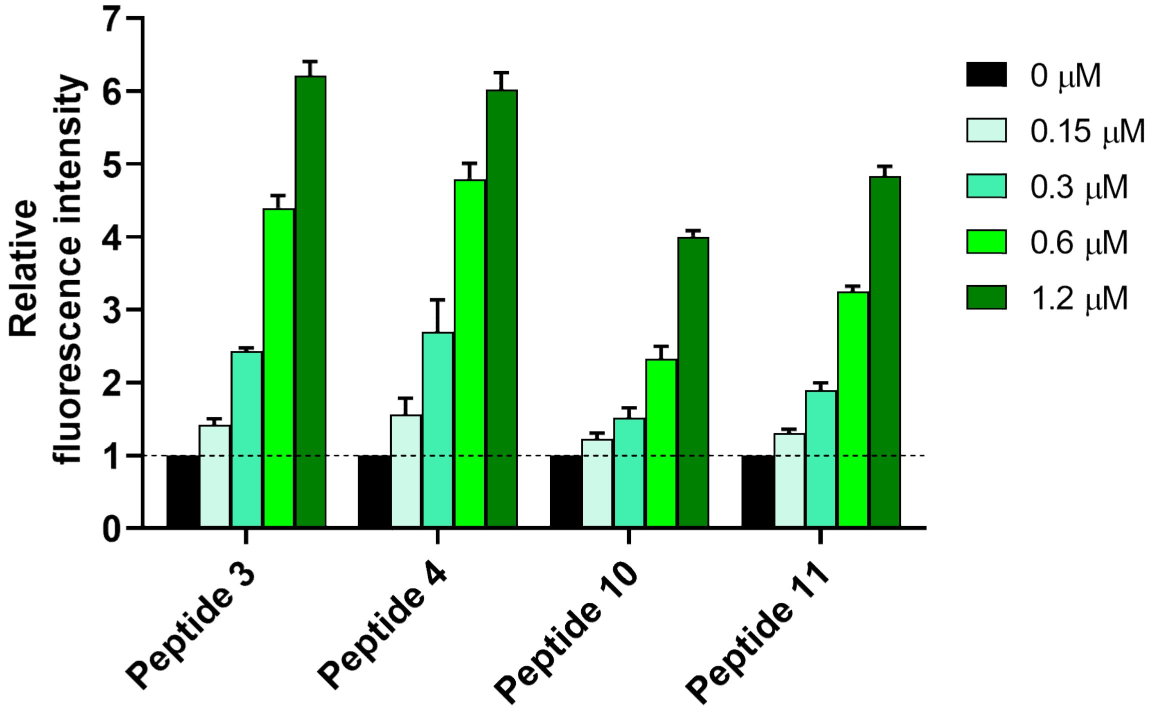

Supplementary Figure S3. Binding ELISA for direct association between peptides and DENV NS1. An ELISA plate was coated with purified DENV-2 NS1 protein and remaining non-specific binding sites were subsequently blocked with BSA. Fluorescent tag-conjugated peptides at the indicated concentrations were incubated in the NS1-coated wells for 1 hour at room temperature. Fluorescent signals were measured by a multiplate reader with a fluorescence mode at the excitation and emission wavelengths of 485 nm and 530 nm, respectively. Relative fluorescence intensity, which represents peptide binding to DENV NS1, was determined by normalization of the fluorescent signals at each peptide concentration with that of a diluent control (0 µM peptide). A dotted line indicates the baseline level of fluorescent signals in the control. Results show mean + SEM of relative fluorescence intensity from 3 independent experiments.

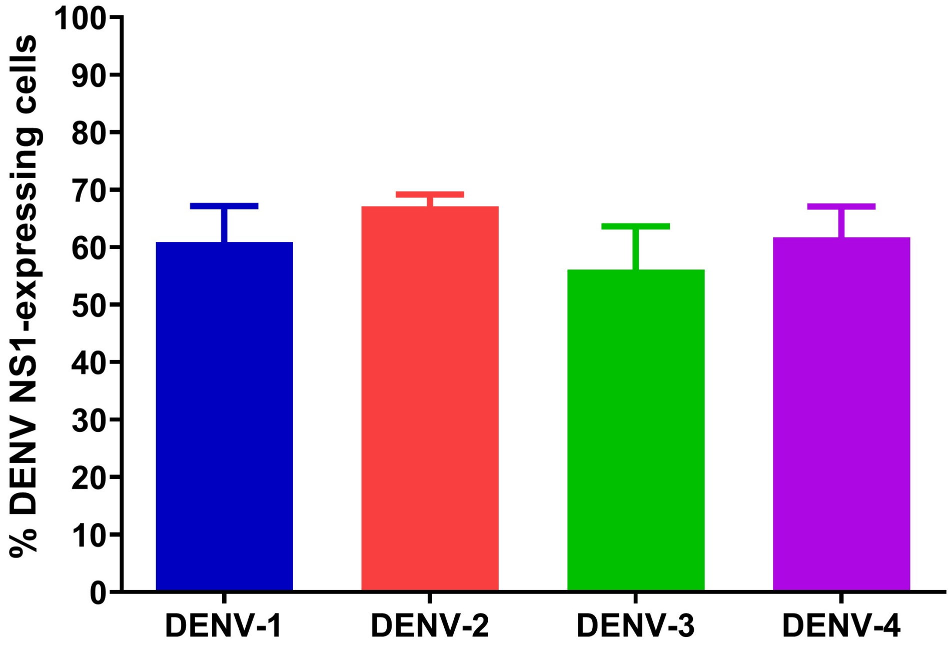

Supplementary Figure S4. NS1 expression in Huh7 cells following infection with all four DENV serotypes. Huh7 cells were infected with DENV-1 (strain Hawaii), DENV-2 (strain 16681), DENV-3 (strain H87), or DENV-4 (H241) at MOIs of 2.5, 0.5, 5, or 1, respectively. At 24-hours post-infection, cells were assessed for the percentage of NS1 expression by immunofluorescence staining and flow cytometry. Results show mean + SEM of % DENV NS1-expressing cells from 3-5 independent experiments.

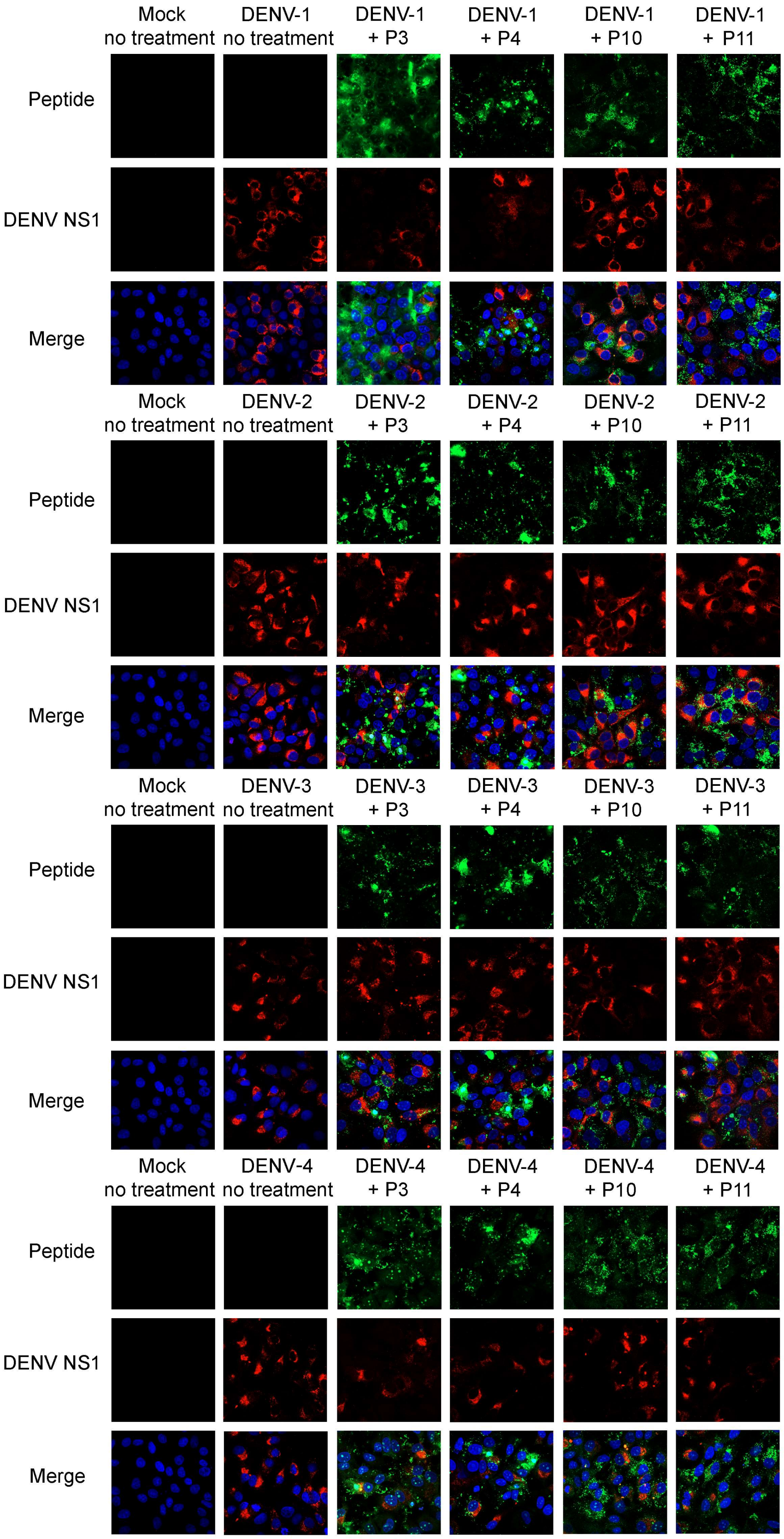

Mock no treatment
DENV-1 no treatment
DENV-1 + P3
DENV-1 + P4
DENV-1 + P10
DENV-1 + P11
Peptide
DENV NS1
Merge
Mock no treatment
DENV-2 no treatment
DENV-2 + P3
DENV-2 + P4
DENV-2 + P10
DENV-2 + P11
Peptide
DENV NS1
Merge
Mock no treatment
DENV-3 no treatment
DENV-3 + P3
DENV-3 + P4
DENV-3 + P10
DENV-3 + P11
Peptide
DENV NS1
Merge
Mock no treatment
DENV-4 no treatment
DENV-4 + P3
DENV-4 + P4
DENV-4 + P10
DENV-4 + P11
Peptide
DENV NS1
Merge

Supplementary Figure S5. Effects of peptide treatment on DENV NS1 expression in Huh7 cells following infection with all four DENV serotypes. Huh7 cells were uninfected (mock control) or infected with DENV-1 (strain Hawaii), DENV-2 (strain 16681), DENV-3 (strain H87), or DENV-4 (H241) at MOIs of 2.5, 5, 0.5, or 1, respectively. At 4-hours post-infection, cells were non-treated or treated with 20 μM of peptide 3 (P3), peptide 4 (P4), peptide 10 (P10), and peptide 11 (P11). Cells were harvested at 24-hours post-infection and assessed for peptide entry and DENV NS1 expression by immunofluorescence staining and confocal microscopy (LSM800; Carl Zeiss Microscopy, Jena, Germany). Objective lens, 63×; green (5-FAM-conjugate peptide); red (DENV NS1); blue (nucleus).

Supplementary Table S1. DNA sequencing and converted amino acid sequences of selected phage clones from biopanning round 3

Phage clone ID	Nucleotide sequence	Amino acid sequence	Peptide ID
14	ACTCTGTTTTCTAAGCCTTATCCGAATTCTAGTCGT	TLFSKPYPNSSR	Peptide 1
24	ACTCCGATGCATTATCCGGCGACTCCTTCTCCGCAT	TPMHYPATPSPH	Peptide 2
55	CAGTTTGGGCCTGTGTTTACGTGGCTTAATCATGCG	QFGPVFTWLNHA	Peptide 3
77	ACTATTACTAATGCTCCGATTAAGGATCTGACTCCT	TITNAPIKDLTP	Peptide 5
11	CTTACGCCGCATAAGCATCATAAGCATCTTCATGCG	LTPHKHHKHLHA	Peptide 6
28	GATCCTCATGGTAGTCTTTTTCCTCGGACTCATCCT	DPHGSLFPRTHP	Peptide 7
29	ACTCAGTATCCGATTGATGGGGATATTTTTAGGAGG	TQYPIDGDIFRR	Peptide 8
54	CATTTGACTTGGATTCCTTCGGTGGTTCGTAATAGT	HLTWIPSVVRNS	Peptide 9
8	TGGCATTGGGCTTGGTATTCTCCGACGGCGCGGATG	WHWAWYSPTARM	Peptide 11
2	TCTTTTGTTAATCTGTGGACGCCGCGTTATTCTTTG	SFVNLWTPRYSL	Peptide 4
9	TCTTTTGTTAATCTGTGGACGCCGCGTTATTCTTTG	SFVNLWTPRYSL	Peptide 4
15	TCTTTTGTTAATCTGTGGACGCCGCGTTATTCTTTG	SFVNLWTPRYSL	Peptide 4
45	TCTTTTGTTAATCTGTGGACGCCGCGTTATTCTTTG	SFVNLWTPRYSL	Peptide 4
73	TCTTTTGTTAATCTGTGGACGCCGCGTTATTCTTTG	SFVNLWTPRYSL	Peptide 4
1	TGGCATTGGCGGCTTTGGGATGTGCCTGATAATCCT	WHWRLWDVPDNP	Peptide 10
7	TGGCATTGGCGGCTTTGGGATGTGCCTGATAATCCT	WHWRLWDVPDNP	Peptide 10
10	TGGCATTGGCGGCTTTGGGATGTGCCTGATAATCCT	WHWRLWDVPDNP	Peptide 10
18	TGGCATTGGCGGCTTTGGGATGTGCCTGATAATCCT	WHWRLWDVPDNP	Peptide 10
20	TGGCATTGGCGGCTTTGGGATGTGCCTGATAATCCT	WHWRLWDVPDNP	Peptide 10
22	TGGCATTGGCGGCTTTGGGATGTGCCTGATAATCCT	WHWRLWDVPDNP	Peptide 10
25	TGGCATTGGCGGCTTTGGGATGTGCCTGATAATCCT	WHWRLWDVPDNP	Peptide 10
26	TGGCATTGGCGGCTTTGGGATGTGCCTGATAATCCT	WHWRLWDVPDNP	Peptide 10
30	TGGCATTGGCGGCTTTGGGATGTGCCTGATAATCCT	WHWRLWDVPDNP	Peptide 10
34	TGGCATTGGCGGCTTTGGGATGTGCCTGATAATCCT	WHWRLWDVPDNP	Peptide 10
35	TGGCATTGGCGGCTTTGGGATGTGCCTGATAATCCT	WHWRLWDVPDNP	Peptide 10
38	TGGCATTGGCGGCTTTGGGATGTGCCTGATAATCCT	WHWRLWDVPDNP	Peptide 10
39	TGGCATTGGCGGCTTTGGGATGTGCCTGATAATCCT	WHWRLWDVPDNP	Peptide 10
56	TGGCATTGGCGGCTTTGGGATGTGCCTGATAATCCT	WHWRLWDVPDNP	Peptide 10
58	TGGCATTGGCGGCTTTGGGATGTGCCTGATAATCCT	WHWRLWDVPDNP	Peptide 10
60	TGGCATTGGCGGCTTTGGGATGTGCCTGATAATCCT	WHWRLWDVPDNP	Peptide 10
62	TGGCATTGGCGGCTTTGGGATGTGCCTGATAATCCT	WHWRLWDVPDNP	Peptide 10
65	TGGCATTGGCGGCTTTGGGATGTGCCTGATAATCCT	WHWRLWDVPDNP	Peptide 10
66	TGGCATTGGCGGCTTTGGGATGTGCCTGATAATCCT	WHWRLWDVPDNP	Peptide 10
68	TGGCATTGGCGGCTTTGGGATGTGCCTGATAATCCT	WHWRLWDVPDNP	Peptide 10
70	TGGCATTGGCGGCTTTGGGATGTGCCTGATAATCCT	WHWRLWDVPDNP	Peptide 10
80	TGGCATTGGCGGCTTTGGGATGTGCCTGATAATCCT	WHWRLWDVPDNP	Peptide 10
**Total number of clones for DNA sequencing**			**36**

Supplementary Table S2. Structures and properties of DENV NS1-binding peptides conjugated with fluorescent and cell penetrating tags

Compound	Structure	Property
Peptide 3	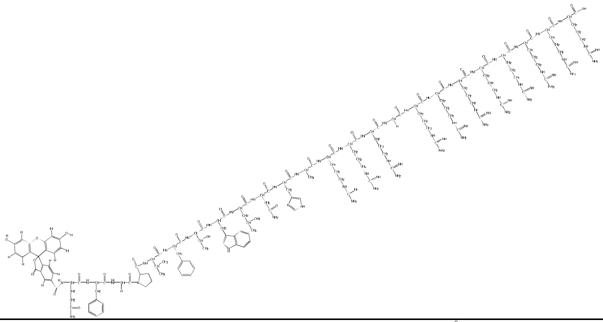	Mass = 3176.66018<br>Mol Formula = $C_{137}H_{227}N_{61}O_{28}$<br>Solubility (mg/mL) = 23.4<br>Formal Charge = 21
Peptide 4	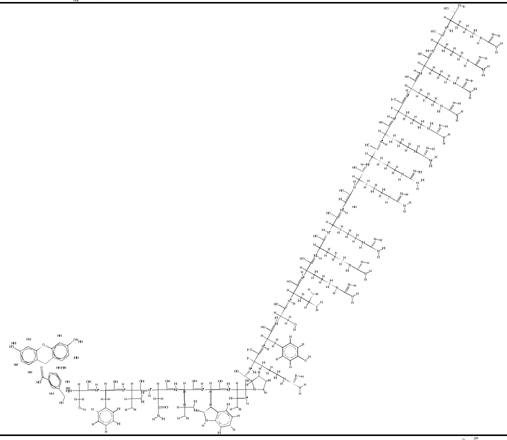	Mass = 3284.107416764<br>Mol Formula = $C_{139}H_{266}N_{62}O_{30}$<br>Solubility (mg/mL) = 25.8<br>Formal Charge = 22
Peptide 10	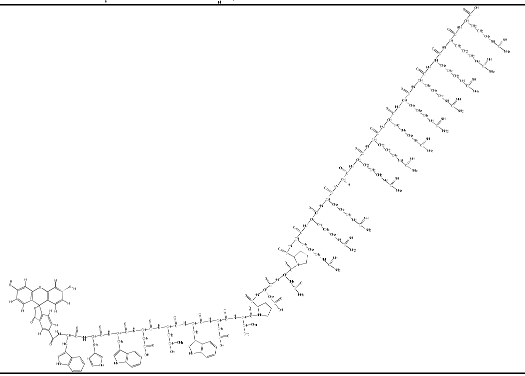	Mass = 3342.8385126499<br>Mol Formula = $C_{143}H_{231}N_{63}O_{32}$<br>Solubility (mg/mL) = 28.2<br>Formal Charge = 26
Peptide 11	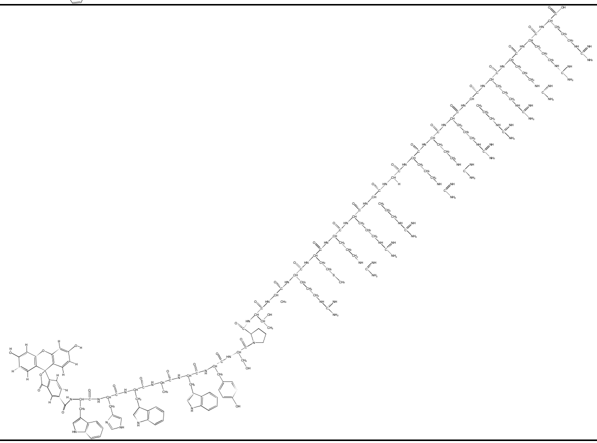	Mass = 3384.8789376766<br>Mol Formula = $C_{145}H_{237}N_{65}O_{29}S$<br>Solubility (mg/mL) = 24.2<br>Formal Charge = 23.4

Supplementary Table S3. Potential binding sites between DENV NS1 and identified peptides with fluorescent and cell penetrating tags

Peptides	Hydrogen bonds on DENV NS1	Hydrogen bonds on peptide	Hydrogen bond length (A^o)	Hydrophobic bonds on DENV NS1	Hydrophobic bonds on peptide	Hydrophobic bond length (A$^{o)}$
Peptide 3 with tags	Glu274	Gln1	2.21	Lys272	Phe2	5.24
	His26	Pro4	2.42	His26	Pro4	3.97
	His26	Val5	2.21	Trp28	Val5	4.25
	His26	Val5	2.17	Trp28	Val5	5.13
	Glu203	Trp8	2.31	Arg324	Phe6	4.54
	Lys214	Leu9	2.15	Arp201	Trp8	4.15
	Glu156	Leu9	2.97	Asp23	Trp8	4.28
	Glu213	His10	1.84	Lys9	Leu9	5.14
				Ala205	His10	3.45
Peptide 4 with tags	Arg324	Ser1	2.28	Lys272	Phe2	4.12
	Arg324	Ser1	2.34	Trp28	Val3	4.25
	Glu274	Ser1	2.25	His26	Leu5	3.80
	Glu274	Phe2	3.12	Lys214	Leu5	4.01
	Arg324	Asn4	2.24	Ile19	Arg9	4.05
	Asn10	Arg9	2.33	Lys9	Tyr11	4.15
	Glu12	Arg9	2.27			
	Trp8	Arg9	2.31			
	Arg192	Leu12	2.11			
Peptide 10 with tags	Glu274	Trp1	2.24	Trp28	Trp6	4.21
	His26	His2	2.18	Trp28	Val8	4.14
	Lys170	Trp3	2.34	Lys9	Val8	4.28
	Arg324	Leu5	2.25	His26	Arg4	4.11
	Glu203	Trp6	2.19	Trp28	Arg4	4.52
	Arg192	Pro9	2.28			
Peptide 11 with tags	Trp28	Trp1	2.21	Trp28	Trp1	5.01
	Trp28	Trp1	2.18	Glu203	Ala4	4.56
	Glu274	Trp3	2.35	Trp8	Tyr6	4.37
	Ser17	Ala4	2.36	Lys11	Ala10	5.34
	Trp8	Tyr6	2.10			
	Trp8	Tyr6	2.34			
	Lys11	Ala10	2.25			
	Glu203	Met12	2.14			
